# Risk of Serious Bleeding with Antiplatelet Therapy for Secondary Prevention Post Ischemic Stroke in Middle East Population

**DOI:** 10.7759/cureus.4942

**Published:** 2019-06-19

**Authors:** Zeinab Awada, Rim Abboud, Samer Nasr

**Affiliations:** 1 Internal Medicine, Lebanese American University-Medical Center, Beirut, LBN; 2 Internal Medicine, Lebanese University, Beirut, LBN; 3 Cardiology, Mount Lebanon Hospital, Beirut, LBN

**Keywords:** ischemic stroke, aspirin, clopidogrel, dual anti platelets, bleeding, secondary prevention

## Abstract

Introduction

Stroke is a devastating disease, causing significant mortality and long-term disability worldwide. Since the bulk of ischemic strokes is attributed to atherothrombosis, secondary prevention with antiplatelet agents is essential to decrease the recurrence of stroke. Aspirin, as well as clopidogrel monotherapy, has been shown to reduce the relative risk of recurrent stroke. However, concerns regarding the efficacy and safety of dual antiplatelet approach still exist. Stroke patients are particularly susceptible to bleeding complications, which might be due to advanced age and comorbidities. Our study assessed the risk of serious bleeding among adult patients on antiplatelet therapy for secondary prevention after stroke who were admitted to Mount Lebanon Hospital (MLH) between 2010 and 2015. It also studied the effect of the antiplatelet therapy, including dose and combination in increasing the risk of bleed.

Methods

A retrospective monocentric study included 454 patients who were admitted for ischemic cerebrovascular accident (CVA) between 2010 and 2015, and discharged on antiplatelet therapy for secondary prevention. Those patients’ records were followed to assess the percentage of patients who developed a major bleed after initiation of antiplatelet therapy.

Results

The risk of serious bleed was highest with aspirin 100 mg monotherapy and dual antiplatelet therapy (DAPT) (Aspirin 100 mg + Clopidogrel 75 mg). Bleeding risk was high during the first three months of therapy. However, the highest risk of bleed exists during the duration extending between three months and one year for both aspirin 100 mg monotherapy and DAPT. Moreover, there was an established relation between patients’ related factors and bleeding risk. Advanced age and smoking were found to contribute to increasing this risk.

Conclusion

Aspirin 100 mg monotherapy and DAPT are associated with the highest risk of bleeding. Although this exists regardless of the duration of antiplatelet therapy, it is highest during the duration extending between three months and one year post initiation of antiplatelet therapy.

## Introduction

Stroke is the fifth leading cause of death in the United States and is a major cause of serious disability for adults [1]. It is defined as an acute loss of neurological function due to abnormal perfusion of brain tissue. According to the World Health Organization, 15 million people suffer from stroke worldwide of which five million die and another five million are permanently disabled. Almost 80% of strokes are secondary to focal cerebral ischemia due to arterial occlusion, and the remaining 20% are due to hemorrhages [2]. Hemorrhagic strokes include intracerebral hemorrhage and atraumatic subarachnoid hemorrhage and result from a rupture of a blood vessel in the brain. Ischemic stroke can be either thrombotic, embolic, or secondary to systemic hypoperfusion.

Although the benefit of antiplatelet agents is uncertain in primary prevention, they are highly recommended for secondary prevention in patients who have experienced a non-cardioembolic stroke or transient ischemic attack (TIA). Patients with history of stroke are at increased risk for subsequent vascular events, including recurrent stroke (highest risk), myocardial infarction (MI), and death from vascular causes - post coronary artery bypass grafting (CABG) or percutaneous transluminal coronary angioplasty (PTCA). The probability of a recurrent stroke after the first one is >3% to 10% in the first month and ≈5% to 14% in the first year which highlights the importance of preventive strategies [3].

Antithrombotic agents are the basis for stroke prevention. Aspirin reduces the relative risk of recurrent vascular events by 13% to 22% and the risk of recurrent stroke by 15% as compared to placebo. Clopidogrel monotherapy has been shown to be superior to aspirin as shown in the CAPRIE (Clopidogrel vs. Aspirin in Patients at Risk of Ischemic Events) trial [3].

According to the American Heart Association/American Stroke Association (AHA/ASA) guidelines released in 2018 for the early management of patients with acute ischemic stroke, all patients with non-cardioembolic acute ischemic stroke (AIS), antiplatelet agents rather than oral anticoagulation are recommended to reduce the risk of recurrent stroke and other cardiovascular events (class 1A) [4]. Moreover, patients presenting with minor stroke, treatment for 21 days with dual antiplatelet therapy (aspirin and clopidogrel) begun within 24 hours can be beneficial for early secondary stroke prevention for a period of up to 90 days from symptom onset (class 2A) as by the AHA/ASA guidelines released in 2014 [5]. Four major antiplatelet drugs have been approved by the Food and Drug Administration (FDA) for prevention of vascular events among patients with a stroke or TIA (aspirin, combination aspirin/dipyridamole, clopidogrel, and ticlopidine). Aspirin by far is the most widely used antiplatelet.

Our study aimed to assess the risk of serious bleed among adult patients on antiplatelet therapy for secondary prevention after stroke who were admitted between 2010 and 2015. It also studied the effect of antiplatelet therapy including dose, combination, and duration of the antiplatelet therapy in increasing the risk of bleeding. Moreover, the effect of age, comorbidities (defined as two or more comorbidities), and smoking were also assessed.

Aspirin

Aspirin reduces the risk of recurrent stroke and other major vascular events by 13% to 22%, according to a meta-analysis of all randomized clinical trials (RCTs) done in ischemic stroke or TIA patients [6]. The efficacy of aspirin was demonstrated in The Chinese Acute Stroke Trial (CAST) and International Stroke Trial (IST). Studies found that acute treatment with aspirin after ischemic stroke reduced the risk for recurrent ischemic stroke by 30% [7].

Clopidogrel

The role of clopidogrel (75 mg daily) in preventing recurrent vascular events in patients who suffered a recent MI, compared with aspirin (325 mg daily) was shown in the CAPRIE study. It showed that long-term administration of clopidogrel to patients with atherosclerotic vascular disease is more effective than aspirin in reducing the combined risk of ischemic stroke, myocardial infarction, or vascular death [8,9].

Dual antiplatelet therapy for secondary stroke prevention and bleeding risk

Several trials and meta-analysis were done that studied the role of dual antiplatelet therapy (DAPT) in secondary prevention and the risk of major bleed. MATCH; Clopidogrel for High Atherothrombotic Risk and Ischemic Stabilization, Management, and Avoidance (CHARISMA); and Secondary Prevention of Small Subcortical Strokes (SPS3) are trials that studied the risk of major bleeding associated with long-term dual antiplatelet therapy after stroke. They showed that the combination of aspirin plus clopidogrel is associated with statistically significant higher bleeding risks than either of the regimens alone [10-12].

Furthermore, the Fast Assessment of Stroke and Transient Ischemic Attack to Prevent Early Recurrence (FASTER) trial and the Effect of Urgent Treatment of Transient Ischemic Attack and Minor Stroke on Early Recurrent Stroke (EXPRESS) study showed a significantly higher risk of symptomatic and asymptomatic bleeds among patients treated with dual antiplatelet therapy, there initiation is accompanied by a doubling of bleeding risk in thefirst30 days[13].

On the other hand, the Clopidogrel in High-Risk Patients with Acute Nondisabling Cerebrovascular Events (CHANCE) trial compared DAPT with aspirin and clopidogrel versus aspirin monotherapy in patients initiated on APT within 24 h of TIA or minor stroke. It showed that the rate of moderate or severe bleeding did not differ between the two groups with superiority of DAPT in reducing the risk of stroke in the first 90 days of initiation of treatment [14].

## Materials and methods

1. Study design

A retrospective monocentric study conducted at Mount Lebanon Hospital between 2010 and 2015.

2. Study population

i. Target population: Patients admitted to Mount Lebanon Hospital (MLH) for ischemic cerebrovascular accident (CVA), from 2010 to 2015, and discharged on antiplatelet therapy for secondary prevention.

ii. Inclusion criteria: All patient above 25 years old with ischemic CVA or TIA discharged on antiplatelet therapy.

iii. Exclusion criteria:

 - Patients with malignancies

 - Patients on anticoagulation therapy

 - Patients with vascular abnormalities

 - Patients with bleeding disorders

3. Ethics

Ethical approval to conduct this study was obtained from the International Review Board of the Faculty of Medical Sciences at the Lebanese University and MLH. Researchers and field workers conducted the study according to the research ethics guidelines laid down in the Declaration of Helsinki.

4. Methods of data analysis

A total number of 454 patients with ischemic CVA were noted between 2010 and 2015. Patients who fitted the inclusion criteria were then selected (338 patients). The medical files of patients were reviewed carefully; every important information was collected including baseline characteristics (Date of birth, sex, lifestyle behaviors: smoking status). The patients were classified into two groups according to the number of comorbidities including hypertension, CVA, diabetes mellitus, peripheral artery disease (PAD), dyslipidemia, coronary artery disease (CAD), and chronic kidney disease (CKD). The first group includes patients with less than two comorbidities, while the other group includes patients with a minimum of two comorbidities. The type of antiplatelet used, dose, and duration of treatment before bleeding were also studied.

All patients who developed symptomatic intracranial bleeding documented on brain imaging (CT scan or MRI) were included. However, patients with gastrointestinal bleeding were included only if major event developed requiring transfusion of 2 or more units of packed red blood cells.

Data were analyzed using the statistical program SPSS (Statistical Package for the Social Sciences) version 18 (IBM Corp., Armonk, NY). Categorical variables were analyzed using frequencies and percentages. Statistical bivariate analysis was performed. To test independence between two dichotomous variables, Pearson’s chi-square test was used. To assess inter-group differences, student T test was used for continuous variables with adequate normal distribution. A p-value < 0.05 was considered statistically significant. A multivariate analysis using logistic regression was carried out for the different doses of the antiplatelet used.

## Results

A total of 454 patients were recruited from which 66 patients were excluded. Fifty patients had incomplete medical files, and 338 patients were included in the study. The mean age of the study participants was 70.69 years (Range 25-99 years) and the majority of the patients (65.7%) had ≥2 comorbidities and 62.7% of the participants were smokers (Table 1).

**Table 1:** Demographic characteristics of study patients *Comorbidities include hypertension, CVA, diabetes mellitus, PAD, dyslipidemia, CAD, and CKD. CVA: Cerebrovascular accident; PAD: Peripheral artery disease; CAD: Coronary artery disease; CKD: Chronic kidney disease.

N	338
Age in years, median (range)	70 (25-90)
Men n (%)	191 (56.5)
Comorbidities* ≥ 2 n (%)	222 (65.7)
Smoker n (%)	212 (62.7)

**Figure 1:**
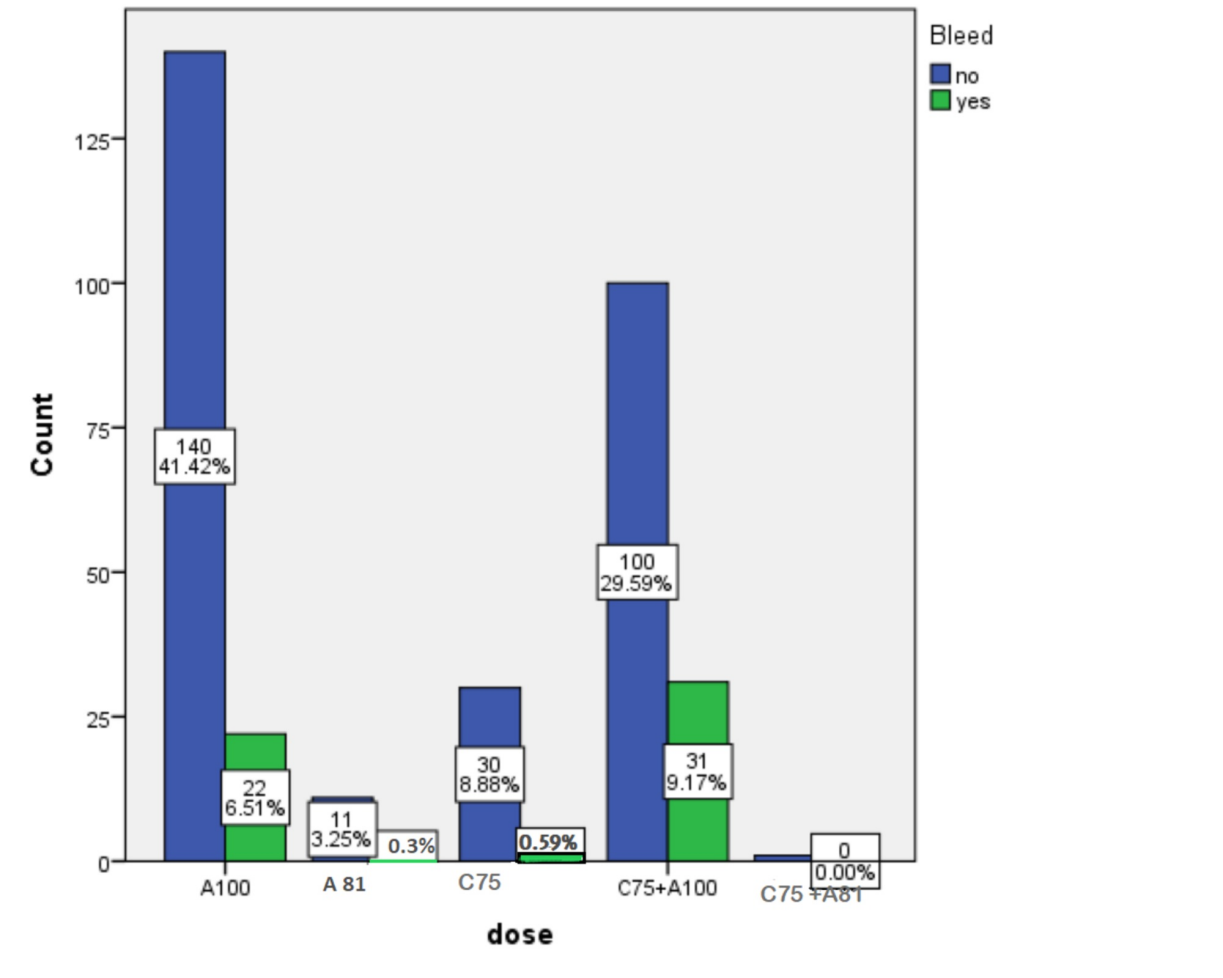
Clustered bar charts for dose of antiplatelet and bleeding A: Aspirin; C: Clopidogrel; A100: Aspirin 100 mg; C81: Aspirin 81 mg; C75: Clopidogrel 75 mg.

Among included patients 16.6% developed a bleed and 82.1% of the those were intracranial bleeding. It is important to note that patients with gastrointestinal bleed were included only if serious bleed occurred which was defined as requiring two or more packed red blood cells (PRBCs). Around 55.36% of those patients who experienced bleed were on DAPT, and 39.28% were on aspirin 100 mg monotherapy. Only 3.57% of those patients had a bleed with clopidogrel monotherapy (Figure 1).

A significant correlation was found between bleeding and aspirin 100 mg (p-value = 0.04) and for the combination of antiplatelet defined as Clopidogrel 75 mg and aspirin 100 mg (p-value = 0.002). However, no relation was established between Clopidogrel 75 mg or aspirin 81 mg and bleeding risk. The results are shown in Table 2.

**Table 2:** The relation between different antiplatelet dosage and bleed The association between different antiplatelet dosage and bleeding is shown using logistic regression model. A score test is used to predict whether or not an independent variable would be significant in the model. P value is statistically significant for aspirin 100 mg monotherapy and DAPT with A100 + C75. A100: Aspirin 100 mg; A81: Aspirin 81 mg; C75: Clopidogrel 75 mg.

Variables (Dose)	Score test*	p-value
A100	3.055	0.04
A81	541	0.462
C75	2.491	0.115
C75 + A100	9.418	0.002

Bleeding with aspirin 100 mg monotherapy and DAPT (aspirin 100 mg + clopidogrel 75 mg) was significant during early treatment duration, and decreased after 12 months of therapy. But 46.4% of patients who experienced bleed had the episode during an intermediate duration of antiplatelet therapy defined between three months and 12 months for both DAPT and aspirin 100 mg monotherapy. The distribution of the bleeding population according to dose and duration of antiplatelet therapy is demonstrated in Table 3.

**Table 3:** Duration and dosage of antiplatelet in patients with bleed A100: Aspirin 100 mg; A81: Aspirin 81 mg; C75: Clopidogrel 75 mg.

Duration	Dose, N (%)				
	C75, 32 (9.5)	A81, 12 (3.6)	A100, 162 (47.9)	C75 + A81, 1 (0.3)	C75 + A100, 131 (38.8)
<3 months	0	0	14.28%	0	17.86%
3-12 months	3.57%	0	17.86%	0	25%
>12 months	0	1.79%	7.14%	0	12.5%

In our study, we assessed patients’ related factors and their contribution to increasing the risk of bleed. The results showed that average age of 75 years increases the risk of a serious bleed in patient on DAPT with significant p-value (0.006) which was assessed using Levene’s test and t-test. In addition, a correlation between comorbidities, smoking and bleeding risk was established. The test results are provided in the table that shows the observed value and the p-value. A significant relationship was found at the 5% risk level between bleeding and comorbidities (p-value = 0.001). About 80.4% of bleeding patients were smokers with a significant p-value of 0.03 between smoking and bleeding risk (Table 4).

**Table 4:** The relation between comorbidities, smoking and bleed

Variable	Pearson Chi-Square value	p-value
Bleed * comorbidities	21.99	0.001
Bleed * smoker	8.928	0.03

## Discussion

Our study showed that the risk of major bleed including gastrointestinal and intracerebral bleed in patients on dual antiplatelet therapy during 2010-2015 for secondary prevention for stroke was 16.6%. Among which 82.1% were intracerebral bleed (ICB) and the rest were gastrointestinal bleed (GIB). This was in contrast to MATCH and SPS3 studies which showed high prevalence of GIB with stable rates of ICB. This can be possibly attributed to the fact that MATCH trial excluded patients with severe comorbid conditions whereas SPS3 trial excluded all patients with a history of intracerebral hemorrhage, modified Rankin score ≥4 (disabling stroke), previous intracranial hemorrhage from non-traumatic causes, and cortical ischemic stroke. The low incidence of GIB in our studied population can be also possibly attributed to the use of gastric protection noticed in 60% of the studied population.

The combination of antiplatelet therapy is highly associated with bleeding risks in our study (p-value = 0.002). However, there was a contribution of aspirin 100 mg and bleeding risk with p-value = 0.04. This was shown in CAST. It demonstrated that acute treatment with aspirin after ischemic stroke reduced the risk for recurrent ischemic stroke by 30% (relative risk reduction) with a small increase of intracerebral hemorrhage [15]. However, no association between bleeding risk and clopidogrel 75 mg monotherapy neither aspirin 81 mg was observed. This shows low bleeding risk with the above regimens. But absolute safety regarding their use can’t be established since only a small percentage of the studied patients was on clopidogrel 75 mg or aspirin 81 mg monotherapy.

There are many trials of major bleeding associated with long-term dual antiplatelet therapy after stroke including MATCH, CHARISMA, and SPS3 trial. These trials showed that the combination of aspirin plus clopidogrel is associated with statistically significant higher bleeding risks than either of the antiplatelet alone. Those results come in concordance with our results, which showed a high risk of serious bleed in patients on DAPT for long term treatment (>12 months) [16].

Other trials have investigated the benefit of a short course of aspirin plus clopidogrel. Combination therapy was shown to be more effective against ischemic events than monotherapy, but results regarding the risk of bleeding in the early phase were contradictory. The FASTER trial showed a significantly higher risk of symptomatic and asymptomatic bleeds among patients treated with dual antiplatelet therapy. But, CHANCE trial showed no difference in the risks of bleeding among both groups [14,16]. Our study has shown a significant risk of bleed with DAPT during the early course of treatment (<3 months). However, results from our study demonstrated the highest risk of bleeding during the duration extending from three months to one year, an intermediate duration that was never assessed before. This risk decreases after 12 months of therapy. The decrease of the risk of bleeding on antiplatelet therapy after 12-month duration can suggest that some form of resistance to antiplatelet drugs might have occurred over time. Yet, the mechanism is unclear and not well investigated.

In this study, we assessed patients’ related factors, and their contribution to increasing the risk of bleeding. The results showed that an average age of 75 years increases the risk of serious bleed in patients on DAPT with significant P value (0.006). In addition, a significant relation between bleeding risk and smoking (p-value = 0.03) has been established. This shows that patient-related factors, mainly age and smoking, not only increase the risk of stroke, but also contribute to increasing the risk of serious bleed in patients on antiplatelet therapy. Thus, aggressive risk-factor management and lifestyle modification advice are essential. Observational studies of patients with a history of stroke indicate that healthy lifestyle behaviors, including regular exercise and abstinence from smoking, are associated with reduced mortality [17].

A strength of our study is the large sample size with a large number of bleeding events. Furthermore, it is the first study done in the Middle East population, mainly the Lebanese one to assess the risk of bleed on antiplatelet therapy for secondary prevention in stroke patients.

Limitations of the study

Our study had several limitations. First, absolute risks of bleeding may have been underestimated because patients at highest bleeding risk were excluded from the trials. Second, some of the data between 2010 and 2011 were missing during our search. Very few of the medical records of the patients were not well filled in terms of medical history and details related to the disease.

Furthermore, it was a retrospective study which may introduce information bias. For example, control of medication intake was not performed therefore; we cannot be sure about the compliance of the patients to their antiplatelet therapy. All our findings were based on documented information mentioned in the medical records, so there was no classification bias, since there was no self-reported data.

## Conclusions

Aspirin 100 mg or combination of antiplatelet can be effective in reducing stroke recurrence, but are associated with high bleeding risk mainly between three months and one year of therapy. However, our study showed that the use of aspirin 81 mg or clopidogrel 75 mg was associated with a lower risk of bleed. Patients’ related factors should always be taken into consideration mainly age, comorbidities, and smoking since there was an established association between the above factors and bleeding risk.

High bleeding risk for stroke secondary prevention should always be taken into consideration whenever initiating antiplatelet therapy. The decision regarding the choice of antiplatelet agent and its duration should be individualized according to the patients’ risk factors weighing the risks and benefits of the chosen treatment.

## Appendices

LIST OF ABBREVIATIONS:

AHA: American Heart Association

AIS: Acute Ischemic Stroke

ASA: American Stroke Association

ASA: Acetylsalicylic Acid

CABG: Coronary Artery Bypass Grafting

CAST: Chinese Acute Stroke Trial

CAD: Coronary Artery Disease

CAPRIE: Clopidogrel vs. Aspirin in Patients at Risk of Ischemic Events

CHARISMA: Clopidogrel for High Atherothrombotic Risk and Ischemic Stabilization, Management, and Avoidance

CVA: Cerebrovascular Accident

DAPT: Dual Antiplatelet Therapy

EXPRESS: Effect of urgent treatment of transient ischemic attack and minor stroke on early recurrent stroke

FASTER: Fast assessment of stroke and transient ischemic attack to prevent early recurrence

FDA: Food and Drug Administration

GIB: Gastrointestinal Bleed

ICB: Intracerebral Bleed

MLH: Mount Lebanon Hospital

MI: Myocardial Infarction

N: Total Population

PAD: Peripheral Artery Disease

PRBC: Packed Red Blood Cell

SPS3: Secondary Prevention of Small Subcortical Strokes Trial

SPSS: Statistical Package for the Social Sciences
